# Streamlined design of a self-inactivating feline immunodeficiency virus vector for transducing *ex vivo *dendritic cells and T lymphocytes

**DOI:** 10.1186/1479-0556-5-8

**Published:** 2007-09-19

**Authors:** Mauro Pistello, Laura Vannucci, Alessia Ravani, Francesca Bonci, Flavia Chiuppesi, Barbara Del Santo, Giulia Freer, Mauro Bendinelli

**Affiliations:** 1 Retrovirus Center and Virology Section, Department of Experimental Pathology, University of Pisa, Pisa, Italy

## Abstract

**Background:**

Safe and efficient vector systems for delivering antigens or immunomodulatory molecules to dendritic cells (DCs), T lymphocytes or both are considered effective means of eliciting adaptive immune responses and modulating their type, extent, and duration. As a possible tool toward this end, we have developed a self-inactivating vector derived from feline immunodeficiency virus (FIV) showing performance characteristics similar to human immunodeficiency virus-derived vectors but devoid of the safety concerns these vectors have raised.

**Methods:**

The pseudotyped FIV particles were generated with a three-plasmid system consisting of: the packaging construct, providing Gag, Pol and the accessory proteins; the vector(s), basically containing FIV packaging signal (*ψ*), Rev responsive element, R-U5 region at both ends, and the green fluorescent protein as reporter gene; and the Env plasmid, encoding the G protein of vesicular stomatitis virus (VSV-G) or the chimeric RD114 protein. Both packaging and vector constructs were derived from p34TF10, a replication competent molecular clone of FIV. The pseudotyped particles were produced by transient transfection in the Crandell feline fibroblast kidney (CrFK) or the human epithelial (293T) cell line.

**Results:**

To broaden its species tropism, the final vector construct was achieved through a series of intermediate constructs bearing a longer *ψ*, the FIV central polypurin tract sequence (cPPT), or the woodchuck hepatitis post-regulatory element (WPRE). These constructs were compared for efficiency and duration of transduction in CrFK or 293T cells and in the murine fibroblast cell line NIH-3T3. Whereas *ψ *elongation and cPPT addition did not bring any obvious benefit, insertion of WPRE downstream GFP greatly improved vector performances. To maximize the efficiency of transduction for ex-vivo murine DCs and T-lymphocytes, this construct was tested with VSV-G or RD114 and using different transduction protocols. The results indicated that the FIV construct derived herein stably transduced both cell types, provided that appropriate vector makeup and transduction protocol were used. Further, transduced DCs underwent changes suggestive of an induced maturation.

**Conclusion:**

In contrast to previously described FIV vectors that were poorly efficient in delivering genetic material to DCs and T lymphocytes, the vector developed herein has potential for use in experimental immunization strategies.

## Background

Upon encountering foreign invaders, dendritic cells (DCs) in the periphery of the body undergo a dynamic and coordinated reprogramming of gene expression, surface phenotype and cellular function [1]. While this maturation is ongoing, DCs migrate to lymphoid organs where they interact with T lymphocytes which, in turn, decode the DC message to start a cascade of events ultimately leading to immune responses against the invading antigens. Thus, at least theoretically, safe and effective systems for delivering antigenic and/or adjuvant proteins/genes to DCs, T cells or both represent valuable means of eliciting and modulating type, extent, and duration of adaptive immune responses [2]. Although initial attempts to achieve this goal using conventional methods were disappointing, recent advances have opened new and more promising avenues [3,4].

Viruses are considered ideal for delivering transgenes due to their inherent ability to bring genetic material into cells but need extensive engineering to overcome limitations such as the spectrum of cells they can enter and the noxious effects they may exert. For example, adenoviral vectors have been shown to be effective at transducing DCs and T lymphocytes [5] but, on a negative side, they have been seen to induce massive production of proinflammatory cytokines and robust vector-specific immune responses [6]. On the other hand, oncoretroviral vectors interfere minimally with normal body and cell functions but are poor at transducing nondividing and rarely dividing cells, such as DCs and resting T cells.

Consistent with the ability of lentiviral genomes to reach the nucleus of host cells even if these do not divide [7], vectors derived from the human immunodeficiency virus (HIV) have been found to transduce DCs and T cells at high efficiency [8-11]. In their current versions, HIV vectors have most of the original viral genome deleted, including some transcriptional elements in the U3 region of the 3' long terminal repeat (LTR) of the DNA used to produce the vector RNA. During reverse transcription, this deletion is transferred to the 5' LTR of the proviral DNA, thus generating two LTRs which are mostly inactive (self-inactivating [SIN] vectors). Also, these vectors are produced using multiple constructs encoding different components to minimize the risk of generating replication-competent viruses.

Because of safety concerns [12], vectors derived from the feline immunodeficiency virus (FIV) are considered a good alternative to the HIV vectors because FIV has never been detected in animal species other than domestic and wild cats and has similar genome organization but minimal sequence homology to HIV, thus minimizing the risk of unwanted recombinations [13].

The FIV vectors described to date have been very successful at delivering transgenes into a variety of cells of different animal species [reviewed in [12]] but have performed poorly when used to transduce DCs, T lymphocytes and non-adherent white blood cells in general [14-16]. In this report, we describe a SIN FIV vector that effectively transduces *ex vivo *murine DCs and T cells.

## Methods

### Parental plasmid and strategy used for packaging and vector construction

**Figure 1:**
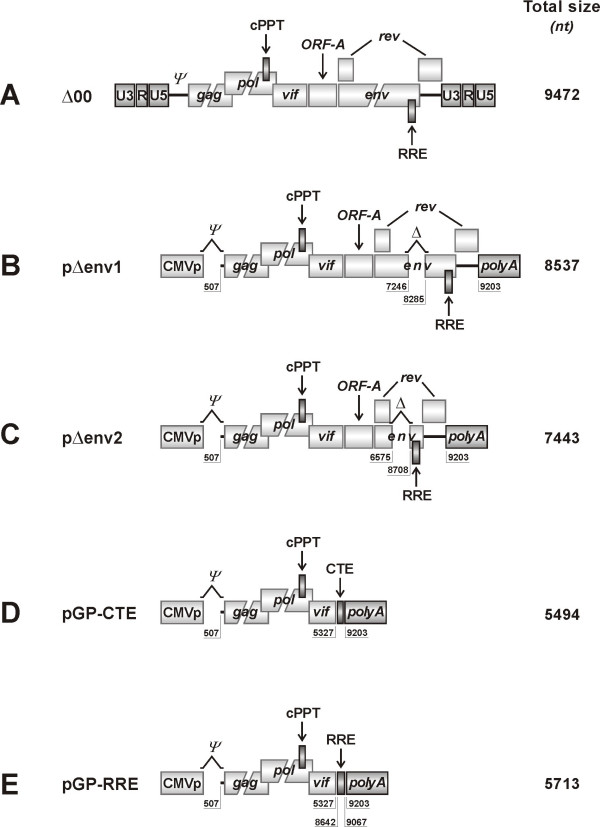
**Schematic representation of the FIV packaging constructs used**. A) Parental clone pΔ00. B) Packaging construct pΔenv1; the 5' and 3' LTRs are replaced by CMVp and bovine growth hormone (BGH) poly A, respectively, *ψ *is partially deleted, and *env *is deleted by an internal 1 Kbp. C) Packaging construct pΔenv2; derived from pΔenv1 by removing the entire *env *except for the terminal ends overlapping the first exon *rev *and the Rev-responsive element (RRE) necessary for nuclear export of unspliced viral RNAs; D) Packaging construct pGP-CTE; obtained by deleting from within *vif *to BGH poly A, and replacing the Rev-RRE system with the Mason-Pfizer monkey virus constitutive transporting element (CTE); E) Packaging construct pGP-RRE; derived from pGP-CTE by replacing the CTE with the RRE retrieved by amplification from the parental clone pΔ00. For this construct, Rev was provided in *trans*.

**Figure 2:**
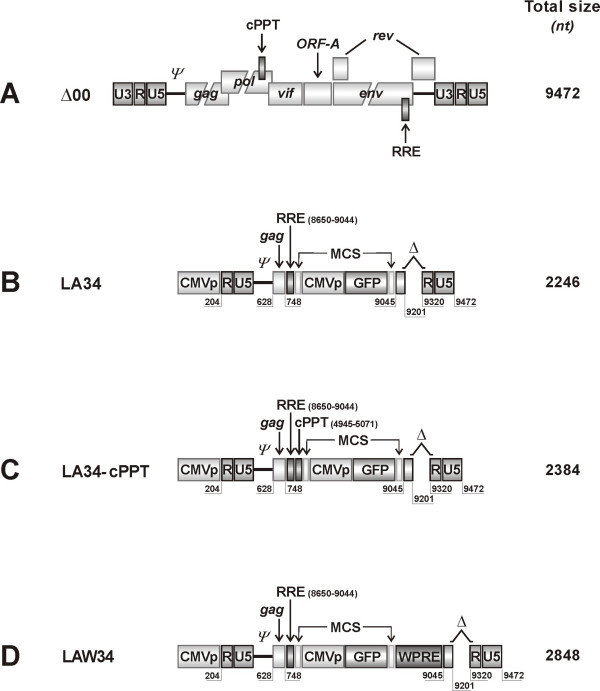
**Schematic representation of the FIV vector constructs used**. A) Parental clone pΔ00. B) Prototype LA34 vector: minimal, self-inactivating vector with both the U3 LTR domains deleted and devoid of all accessory and structural proteins except *ψ*, a 120 nt stretch at the of 5'-end *gag *containing the domains important for RNA encapsidation, and the RRE. MCS, the multiple cloning site, contains the CMVp-GFP cassette used as reported gene for all vectors. C) LA34-cPPT, vector derived from LA34 by inserting the central poly-purine tract (cPPT), important for nuclear import of the FIV preintegration complex, between RRE and MCS. D) LAW34, vector obtained by inserting the woodchuck post-trascription regulatory element (WPRE) in LA34 downstream MCS. Variant vectors having a longer (310 nt) *gag *fragment (LA34-L and LAW34-L, respectively) were also produced but are not shown. Total size indicates the number of nucleotides of the vector without the CMVp-GFP cassette.

Prototype vectors and packaging construct were developed from pΔ00 (Fig. 1A and 2A), a replication-competent molecular clone of the Petaluma strain of FIV (FIV-Pet), derived from plasmid p34TF10 [GenBank: NC_001482] and produced in our laboratory by substituting a tryptophan codon for the stop codon in the accessory gene ORF-A [17]. As reported [18], an intact ORF-A is essential for optimal FIV replication in lymphoid cells. Nucleotide (nt) positions of packaging and vector constructs are referred to the NC_001482 sequence. The sequence of primers used in polymerase chain reaction (PCR) is available by e-mail on request. All the intermediate and final constructs described below were checked for proper insertions and absence of unwanted mutations by cycle sequencing using an automated DNA sequencer (GE Healthcare, Milan, Italy). All vectors were tested using enhanced green fluorescent protein (GFP) as reporter molecule.

#### Packaging constructs

Four packaging constructs were developed and tested. Due to minimal activity of the FIV LTR in non feline cells [19], all constructs had the 5' and 3' LTRs of pΔ00 replaced with cytomegalovirus early promoter (CMVp) and bovine growth hormone poly A, respectively. Also, deletion of the 5'LTR was extended to nt position 507 to remove most of the RNA packaging site (*ψ*). From this intermediate construct the packaging constructs were produced as follows:

*pΔenv1*, retaining the Rev and Rev-responsive element (RRE), necessary for the nuclear export of Gag-Pol mRNA, and containing an internal 1,044 nt deletion within *env *(nt position 7246–8289) obtained by digestion with the restriction enzymes *Bcl*I and *Spe*I (New England Biolabs, Celbio, Milan, Italy), filling up the protruding ends with Klenow DNA polymerase (New England Biolabs) and joining of resulting blunt-ends with T4 DNA ligase (Fermentas, M-Medical, Milan, Italy) (Fig. 1B).

*pΔenv2*, retaining the Rev and RRE but with the internal *env *deletion extended from end of the first exon Rev to beginning of RRE (nt position 7246–8289). This deletion was created by PCR using overlapping primers (Fig. 1C).

*pGP-CTE*, containing a truncated *vif *and deleted of ORF-A, *env*, *rev *and RRE. This genome portion was removed by digestion with the restriction enzymes *Bsp*MII (nt position 5327) and *Blp*I (nt position 9203). The Rev/RRE system was replaced by introducing the Mason-Pfizer monkey virus constitutive transport element (CTE) downstream *vif *(Fig. 1D).

*pGP-RRE*, having the same deletion as *pGP-CTE *and containing RRE (nt position 8642–9067), retrieved by PCR from pΔ00, in place of CTE (Fig. 1E). The Rev/RRE system was restored by providing Rev in trans.

#### Vector constructs

The following vector constructs were developed and tested:

*LA34*, produced from pΔ00 by sequential steps as follows: the U3 region of the 5' LTR (nt position 1–203, as referred to NC_001482) was replaced with pCMV amplified from pcDNA3.1 plasmid (Invitrogen Life Technologies, Milan, Italy) by digestion with the restriction enzymes *Psh*AI and *Sac*I (New England Biolabs). To prevent LTR regeneration during reverse transcription, an internal 120 bp segment of U3 in the 3'LTR (nt position 9201–9320), containing the cis-acting transcriptional elements AP-1, AP-4 and ATF-binding sites and TATA box [20], was also removed by PCR using overlapping primers. The same strategy was applied to delete the region from nt position 749 to 9045, encompassing most of the *gag *and the entire *pol *and *env *genes. Finally, the *env *segment containing RRE (nt position 8650–9038) was inserted downstream the *gag *stretch together with a multiple cloning site (MCS) containing *Asu*II, *Cla*I, *Sac*II, *Blp*I, *Kpn*I, and *Pac*I restriction sites, and used herein for cloning the reporter GFP gene (Fig. 2B).

*LA34-cPPT*, obtained by inserting the central poly-purine tract (cPPT, nt position 4945–5071) that in FIV is localized within *pol *and is important for nuclear import of the preintegration complex (PIC) [21], in LA34. The cPPT was retrieved by PCR from pΔ00 and inserted between RRE and MCS (Fig. 2C).

*LAW34*, obtained by inserting the woodchuck hepatitis post-transcriptional regulatory element (WPRE; kind gift of Dr. Stefano Indraccolo, University of Padua, Italy) in LA34 downstream MCS (Fig. 2D).

*LA34L and LAW34L*, variants of the above LA34 and LAW34, respectively, in which the *gag *stretch was extended from the original 120 nt (nt position 628–747) to 310 nt (628–937) by a two step PCR. They were prepared because reports [22,23] have suggested that, in FIV, *gag *domains downstream the main *ψ *determinants may contribute to efficient RNA encapsidation.

### Other plasmids

The Rev provided in *trans *to the *pGP-RRE *was obtained by amplifying the corresponding mRNA from RNA of FIV-Pet infected Crandell feline kidney fibroblast (CrFK) cells. The cDNA was then cloned into the pcDNA3.1 plasmid (pcDNA-Rev). Constructs pcDNA-Rev and *pGP-RRE *were cotransfected at equimolecular ratio. FIV particles were pseudotyped with the vesicular stomatitis virus glycoprotein envelope (Env) VSV-G (495 amino acids [aa]) or the chimeric retrovirus glycoprotein RD114/TR (546 aa) [24]. VSV-G was encoded by pCMV-VSV-G derived from the pcDNA3.1 plasmid and RD114/TR by the phCMV-RD114/TR plasmid (kind gift of Dr. François-Loic Cosset, Ecole Normale Supérieure, Lyon, France).

### Cell lines and primary murine cells

The cells used included the CrFK, highly permissive to FIV-Pet, and the human epithelial 293T and murine fibroblast NIH-3T3, two nonfeline lines that do not permit FIV replication. All cells were propagated in Dulbecco's modified Eagle medium (D-MEM, Sigma-Aldrich, Milan, Italy) supplemented with 10% fetal calf serum (FCS), 100 U/ml penicillin, 100 μg/ml streptomycin and 2 mM L-glutamine (Sigma-Aldrich), at 37°C in 5% CO_2_. Murine DCs were generated from the bone marrow (BM) of 6- to 10-week old BALB/c mice. Briefly, BM cells were flushed from the femurs, filtered through a 200 μm mesh to remove fibrous tissues, and cleared from erythrocytes with ammonium chloride. Residual cells were cultured at 2 × 10^6 ^cells/ml in complete RPMI 1640 medium (Sigma-Aldrich), supplemented with 10% FCS, penicillin-streptomycin and glutamine, and induced to differentiate into DCs with 20 ng/ml recombinant mouse granulocyte macrophage colony-stimulating factor (GM-CSF) and 5 ng/ml recombinant mouse interleukin (IL)-4. Floating cells were removed, and fresh GM-CSF/IL-4 enriched medium was added at days 3 and 5 of culture. On day 7, non-adherent and loosely adherent DCs were collected and analyzed by flow cytometry. While control cells cultured with no GM-CSF and IL-4 were essentially negative, they were 55% CD11c-positive, 70% CD80-positive and 15% CD40-positive, thus exhibiting the expression profile of immature DCs [25]. Microscopic examination of the cultures also revealed that they were rich in cells with DC morphology. Murine T lymphoblasts were produced by concanavalin A stimulation of spleen cells from the same mice. Briefly, spleen cells were cultured at 2 × 10^6^/ml in 6-well plates for 5 days in complete RPMI 1640 medium supplemented with 50 unit/ml IL-2, 10% FCS, penicillin-streptomycin, and glutamine. These cells were 30% CD4 positive and 12% CD8 positive.

### Vector production

Vectors were generated in CrFK or 293T cells. Briefly, 2.8 × 10^6 ^cells were seeded in 10 cm Petri dishes and one day later co-transfected with one of the vector plasmids, pΔenv1, and either the VSV-G or the RD114/TR Env (4:5:1; 20 μg total DNA) using a modified calcium phosphate method [26]. Transfection efficiency was evaluated 48 h later by counting GFP-positive cells by flow cytometry with a FACScan and a CELLQuest Version 2 software (BD Biosciences, Milan, Italy). Vector content in the culture fluids collected on the same day was determined by measuring FIV p25 capsid protein and the number of FIV RNA genome copies as previously described [26,27], following clarification at 1,500 rpm for 10 min and 0.45 μm filtration. Supernatants were aliquoted in 1 ml volume and stored at -80°C until use.

### Standard transduction protocol

The day before transduction, 24-well plates were seeded with 7 × 10^4 ^293T cells, 5 × 10^4 ^CrFK cells, 5 × 10^4 ^NIH3T3 cells, 5 × 10^5 ^DCs, or 2 × 10^6 ^T lymphoblasts per well in 1 ml complete medium. Eighteen h later, the medium was replaced with the same volume of vector suspension. Transduction efficiency was evaluated by counting GFP positive cells by flow cytometry 2 days post-transduction (PT).

### FIV vector titer

Vector titers were determined in 293T cells and expressed as number of transduction units (TU) per ml. Briefly, 7 × 10^4 ^cells were transduced as described above with the vector preparation under test serially diluted 10-fold in culture medium. Two days later, the cells were harvested and analyzed for GFP fluorescence by flow cytometry. Each dilution was tested in triplicate.

### FIV vector safety evaluation

**Figure 3:**
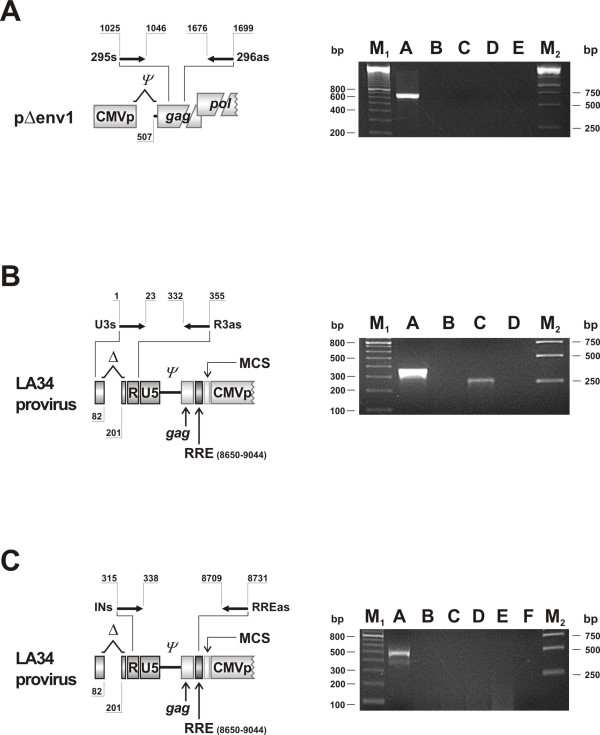
**Safety evaluation of packaging and vector constructs**. A) pΔenv1 transportation from transfected to transduced cells as evaluated by *gag *p25 PCR using the indicated primers. Lane A: DNA from transfected 293T cells; lane B: no template control; lanes C and D: DNA and RNA from transduced 293T cells; lane E: DNA from mock transfected cells. B) Translocation of the U3 deletion to the 5'LTR, as checked by PCR using primers annealing to the beginning U3 and within the R region of LA34 proviral DNA. Lane A: pΔ00 plasmid (full-length LTR), lane B: no template control; lane C: DNA from transduced 293T cells, lane D: DNA from mock transduced cells. C) Analysis of LTR directed transcription as tested by PCR using primers upstream the GFP promoter. Lane A: DNA of transduced 293T cells; lane B: no template control; lanes C and D: RNA from transduced 293T cells with and without DNase treatment prior to reverse transcription; lanes E and F: RNA from mock transduced cells treated as for C and D. Primer nt position referred to the NC_001482 sequence. M_1_: 100 base-pair ladder, M_2_: Gene ruler 1 Kb DNA ladder (GE Healthcare).

Nucleic acids in supernatants, collected from transfected 293T cells and treated as described under "Vector production", were extracted using the QIAamp Viral RNA kit (Qiagen, Milan, Italy). Genomic DNA and RNA from 6 × 10^4 ^transfected or transduced 293T cells were extracted with QIAamp DNA Blood Kit and RNeasy kit (Qiagen), respectively. Viral and genomic RNAs were treated with RNase-free DNase (Qiagen) to eliminate residual DNA. Presence of pΔenv1 plasmid and RNA transcripts in supernatants and transduced cells was investigated by PCR using 295s-296as primers targeting FIV p25 capsid protein (Fig. 3A). Translocation of the U3 deletion from the 3' to the 5'LTR in the vector provirus was examined by amplifying genomic DNA from transduced cells with U3s-R3as primers (Fig. 3B). Inactivation of LTR mediated transcription was ascertained by amplifying cDNA of transduced cells with INs and RREas primers (Fig. 3C). Reverse transcription was carried out with an avian myeloblastosis virus reverse trascriptase (RT) (Finnzyme, Celbio, Milan Italy) and the specific antisense. Amplification profiles were as follows: initial denaturation 94°C 2 minutes; cycling 94°C 30 seconds, 60°C (54°C for 295s-296as) 30 seconds, 72°C 30 seconds (40 seconds for 295s-296as), 35 cycles; extension 72°C 10 minutes. The 5'LTR amplicon was cycle sequenced using the automated ALF ExpressII DNA sequencer (GE Healthcare, Cologno Monzese, Italy).

## Results

### Genome organization of the packaging and vector constructs

Packaging and vector constructs were both derived from pΔ00, a replication competent clone of FIV-Pet (Fig. 1A and 2A).

The packaging constructs provided Gag and Pol and were under the control of the CMVp. Basically, they lacked the untranslated 5'LTR-*gag *region that, together with the initial part of *gag*, form the *ψ *signal required for viral RNA encapsidation into assembling virions and were deleted of part (*pΔenv1 *and *pΔenv2 *constructs) or the entire (*pGP-CTE *and *pGP-RRE*) *env*. The latter constructs also lacked Vif, ORF-A, and Rev. Since nuclear export of unspliced (i.e. Gag-Pol encoding mRNA) and singly spliced mRNAs occurs through Rev binding to the RRE motif, in *pGP-CTE *the Rev/RRE system was replaced by a CTE and in *pGP-RRE *RRE was maintained and Rev provided in *trans *by cotransfection of pcDNA-RRE plasmid (Fig. 1B–1D).

The SIN vector LA34 (Fig. 2B) was under the control of the CMVp and had few remnants of the FIV genome, namely 1) the *ψ *signal, 2) the RRE motif, placed downstream *ψ *and interacting with the Rev provided by the packaging construct or pcDNA-RRE, 3) the untranslated region between *env *and 3'LTR, and 4) the two LTRs, both deleted of the U3 domain to avoid the generation of functional LTRs during reverse transcription. Due to extensive rearrangement and deletions, LA34 was 2.2 Kb in size.

### Packaging and vector constructs are safe and stable

The packaging constructs (Fig. 1B–1E) were developed and tested for stability and safety (non-infectivity) in CrFK and 293T cells. Briefly, the packaging constructs were transfected into cells which were propagated for at least two weeks and monitored twice a week for FIV p25 and viral RNA release into supernatant. Further, cell-free supernatants were collected at 7 and 14 days post-transfection, and seeded into fresh CrFK cell that were cultivated for additional two weeks. Except for a low and transient production of p25 found two-three days post-transfection, neither FIV RNA nor infectious particles were found in supernatants (data not shown). The results indicated that the packaging constructs were free from residual pΔ00 plasmid molecules, were stable, and did not generate infectious virus.

Transduction efficiency, stability, and safety were tested in CrFK and 293T cells, using LA34 pseudotyped with VSV-G (LA34/VSV-G) generated in CrFK or 293T cells with pΔenv1 as packaging construct. The proportions of GFP-positive CrFK and 293T cells 2 days post-transfection were generally greater than 75%. As determined by measuring p25 and number of vector RNA copies in the supernatant from day 2 to 7 post-transfection, LA34/VSV-G production peaked at day 2 or 3 (data not shown). Vector particles collected at these times were pelleted by ultracentrifugation and analyzed by western blot for protein content. Regardless of whether produced in CrFK or 293T cells, the vector generated protein patterns that, with the obvious exception of Env, were identical to the one of whole FIV-Pet virus used as control, indicating that proper generation and maturation of virions had taken place (data not shown). As shown in Table 1, vector titers exceeded 10^9 ^RNA copies/ml and 10^6 ^TU/ml in 293T cells and were slightly higher when the vector was produced in CrFK rather than 293T cells.

**Table 1:** Vector titers generated from transfected 293T or CrFK cells^a^

**Vector/Env used for pseudotyping**	**Vector produced in**	**Vector titer (RNA copies/ml)**	**Concentration**	**Transduction units/ml**^b^
*LA34/VSV-G*	CrFK	8.5 × 10^9^	None	3.8 × 10^6^
	293T	1.8 × 10^9^	None	1.2 × 10^6^
*LAW34/VSV-G*	293T	5.0 × 10^9,c^	None	2.5 × 10^7^
		2.1 × 10^10,c^	Ultracentrifugation	3.0 × 10^7^
	293T	7.0 × 10^9^	PB^d^	3.0 × 10^7^
*LAW34/RD114/TR*	293T	1.4 × 10^10,c^	Ultracentrifugation	6.0 × 10^7^
		4.0 × 10^9,c^	PB	1.2 × 10^8^

^a ^Titers measured in supernatants collected 2 days post-transfection, average of three independent experiments.

^b ^Measured in 293T cells.

^c ^Same supernatant, titrated as such or after 10 fold concentration by ultracentrifugation.

^d ^Polybrene.

Vector safety was evaluated by several approaches. First, the progeny particles were checked for pΔenv1 incorporation by testing them as well as transduced cells for a gag p25 sequence contained in the packaging construct only (Fig. 1B to 1E). The 674 bp amplicon generated by the PCR and RT-PCR assays used was readily detected in the DNA of transfected cells (positive control) but uniformly absent in the vector particles (not shown) and in transduced cells (Fig. 3A). Second, it was checked whether the U3 deletion, created in the 3'LTR of the vector construct, was indeed translocated to the 5'LTR of proviral DNA by using primers that generated amplicons of different sizes from the full-length (352 bp) and the U3 deleted LTRs (232 bp). The amplicon generated from the DNA of transduced cells was clearly smaller than the one generated from the pΔ00 plasmid used as a source of full-length LTR (Fig. 3B) and had the sequence expected for the deleted LTR (not shown). Third, functional inactivation of the 5' LTR was checked by examining transduced cells for LA34 RNA genomes, the transcription of which would have required a full-length 5'LTR. As shown by Fig. 3C, while the DNA of transduced cells was clearly positive for LA34 sequences, the RNA obtained from the same cells was uniformly negative, regardless of whether it was digested or not with RNase-free DNase. Collectively, these findings demonstrated that LA34 is indeed a SIN vector, that the pseudotyped particles it generates are safe, and that no vector RNA is produced by LA34 transduced cells.

### LA34 is best packaged by pΔenv1 and preferentially transduces feline cells

The packaging constructs were tested for ability to produce LA34/VSV-G virions in 293T cells that were transfected with equimolecular amounts of packaging, vector, and VSV-G plasmids. Supernatants collected 2 days post-transfection were analyzed for virus release by measuring p25 and vector RNA genome copies and for competence for transduction in 293T cells. The efficiency of transfection was similar for all plasmid combinations and averaged 80%. As shown in Table 2, LA34/VSV-G production with pΔenv1 and pΔenv2 was very high, as shown by the high levels of p25 produced, 10^9 ^vector RNA copies and 10^6 ^TU/ml. In contrast, both vector RNA and TU titers of VSV-G pseudotyped particles produced by using pGP-RRE and pGP-CTE were one and two logs lower, respectively, suggesting that virus release, rather than infectivity, was less efficient. The pseudotyped particles generated with pΔenv1 and pΔenv2 were further tested for transduction in CrFK. Since the LA34/VSV-G generated with pΔenv1 performed slightly better in this cell subtype, this construct was selected as packaging for subsequent experiments (data not shown).

**Table 2:** LA34/VSV-G pseudotyped particles generated 2 days post-transfection in 293T cells^a^

**Packaging construct used for vector production**	**p25 optical density**	**Vector RNA copies/ml**	**Transduction units/ml**^b^
*pΔenv1*	2.24	9.3 × 10^9^	6.4 × 10^6^
*pΔenv2*	>2.50	3.0 × 10^9^	3.4 × 10^6^
*pGP-CTE*	1.09	2 × 10^6^	1.0 × 10^3^
*pGP-RRE*^c^	1.92	8.1 × 10^8^	3.0 × 10^5^

^a ^Average of three independent experiments, mean efficiency of transfection 80% (range 75–90%);

^b ^Titer measured in the indicated cells 2 days post-transduction;

^c ^Rev provided in *trans *by cotransfection of pcDNA-Rev.

**Figure 4:**
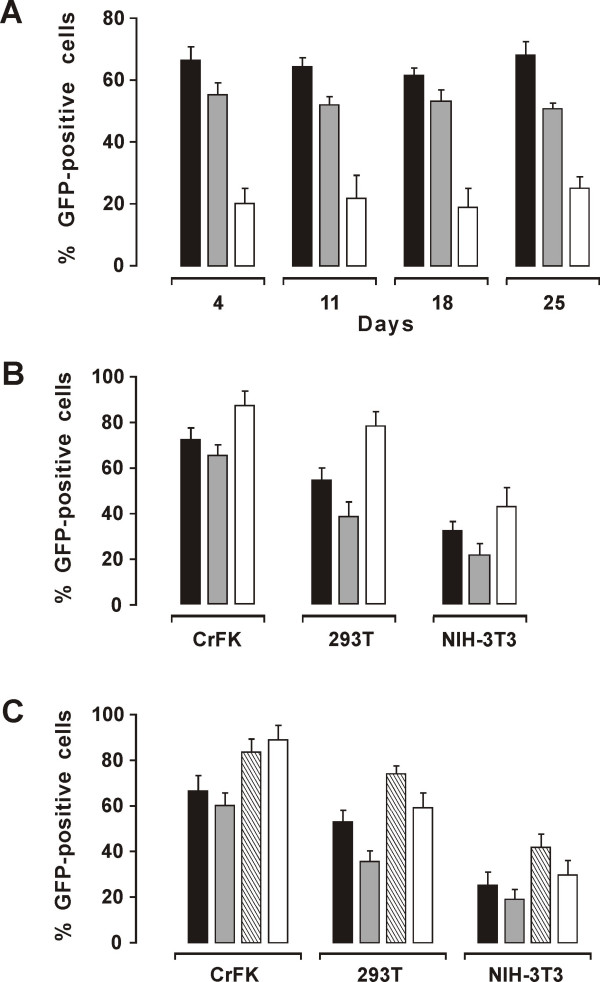
**Efficiency and duration of transduction by naïve and variously modified LA34/VSV-G as determined in CrFK, 293T and NIH-3T3 cells**. A) Efficiency and stability of transduction in CrFK (solid columns), 293T (shaded columns) and NIH-3T3 cells (empty columns) as evaluated by flow cytometry at the indicated times PT. B) LA34-cPPT (shaded columns), LAW34 (empty columns) and LA34 (solid columns). Percent GFP-positive cells evaluated by flow cytometry 4 days PT. C) LA34-L (shaded columns) and LAW34-L (empty columns) versus LA34 (solid columns) and LAW34 (striped columns). Percent GFP-positive cells evaluated by flow cytometry 4 days PT. Bars represent the standard deviation as calculated from three independent experiments.

Transduction efficiency and duration of transgene expression were assessed by inoculating LA34/VSV-G produced in 293T cells into CrFK, 293T and NIH-3T3 cultures at varying RNA copy numbers. The best results were obtained with 10^9 ^copies, corresponding roughly to 10 TU/cell, which at the first readout, 4 days PT, transduced 66% and 52% of CrFK and 293T cells, respectively. In contrast, GFP-positive NIH-3T3 cells rarely exceeded 20%, indicating that these cells were largely refractory to transduction by this vector. Furthermore, during 25 days of observation, GFP-positive cell numbers remained essentially unchanged regardless of initial transduction level, indicating that transduction and transgene expression were stable, as a likely result of vector integration into the cell genomes (Fig. 4A). No infectious virus was detected in the transduced cultures at any time, not even after passaging the culture fluids in fresh CrFK cells repeatedly (data not shown).

### Insertion of the post-transcription regulatory element WPRE increases LA34 transduction efficiency for NIH-3T3

Because the findings above were indicative of a preferential ability of LA34 to transduce feline CrFK cells relative to the non feline cells 293T and NIH-3T3, we made efforts to widen its breadth of action by improving nuclear translocation of PIC, stabilization of transgene mRNAs, and incorporation of vector RNA into pseudotyped particles that are major determinants of lentiviral cell transduction and transgene expression [28]. Two short single-stranded regions of the lentiviral genome DNA (flaps) are believed to optimize viral genome folding, thus enhancing its steric fit in the nuclear pore [29]. In FIV, one of these flaps is located upstream the 3' LTR (U3PPT) and the other close to the 3' end of *pol *(central PPT, cPPT) [21]. Since LA34 contains only the U3PPT, we inserted the cPPT between RRE and MCS (construct LA34-cPPT, Fig. 2C), similar to what already done in the FIV vectors developed in previous reports [28,30]. However, the change had no appreciable effects on vector performance in any of the three cell lines (Fig. 3B).

When we inserted the RNA transport WPRE element downstream of MCS in LA34 (LAW34, Fig. 2D), so that it could be incorporated into transgene mRNA, the efficiency of transduction improved greatly. Compared to LA34, LAW34 showed similar RNA titers but the TUs per ml were approximately 1 log higher (Table 1). Most importantly, LAW34/VSV-G gave rates of NIH-3T3 cell transduction that were nearly twice as great. In fact, close to 50% of the latter cells expressed GFP and did so for at least 4 weeks (Fig. 4B and data not shown).

Recent reports have suggested that the main packaging domain in the *gag *of FIV is comprised in a 120 nt stretch [31]; however, previous studies had suggested the existence of additional encapsidation determinants in *gag*, bringing *ψ *to about 300 nt in size [23]. We, thus, also constructed versions of LA34 and LAW34 having a 311 nt-long *ψ *(LA34-L and LAW34-L). When compared to the respective vectors with 120 nt-long *ψ*, these versions showed no appreciably increased titers (data not shown) and, with the exception of LAW34-L for CrFK cells, had reduced transduction efficiencies (Fig. 4C). As a result of these studies, LAW34 was selected for the experiments below.

### Effect of transduction protocol on LAW34 efficiency

**Figure 5:**
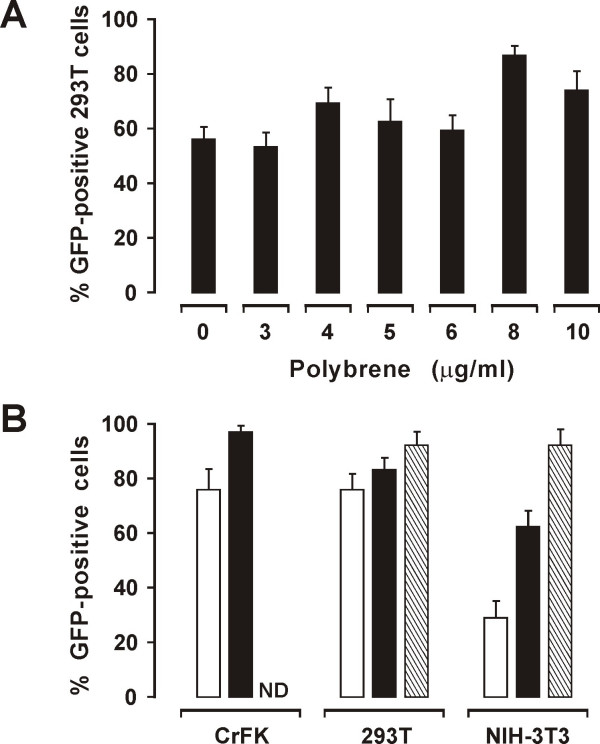
**Evaluation of three transduction protocols**. A) Effect of PB at the indicated concentrations on LA34/VSV-G transduction of 293T cells. B) Effect of using the standard (empty columns), PB (solid columns) or PB/DT protocol (striped columns) on LA34/VSV-G transduction of the indicated cells. Bars and percent GFP-positive cells evaluated as in Fig. 4. ND, not done.

In these experiments, we compared the standard transduction method described under Methods with several protocols that have been shown to increase transduction efficiency by other vectors. These included: 1) vector ultracentrifugation, a procedure frequently used to concentrate VSV-G pseudotyped viruses that, unlike lentiviral Env-coated pseudoparticles, do not tend to shed Env [32]; 2) low-speed centrifugation following poly-L-lysine addition [33]; 3) addition of polybrene (PB), a polycation that forms large complexes with viral particles leaving out nonviral proteins and other factors that may be inhibitory (protocol PB) [34]; 4) treating the cells with the vector twice, four h apart (double transduction; protocol DT); and 5) combined use of 3 and 4 (protocol PB/DT). LAW34/VSV-G produced in 293T cells (5 × 10^9 ^vector RNA copies/ml) was concentrated 10-fold by ultracentrifugation at 200,000g at 4°C for 2 h. In spite of a 4-fold increment in vector RNA titer, transduction efficiency was essentially unchanged relative to the standard method (Table 1). The same output was observed by using protocol 2 in which the virus was concentrated by aggregation with poly-L-lysine. Moreover, the use of poly-L-lysine often caused shrinking, granulation, and, occasional detachment of the cells (data not shown). Protocols 3 to 5 were instead beneficial. A dose-response study of PB in 293T using a vector RNA copy/cell ratio of 200/1 cells (approx. 10 TU/cell) yielded the highest transduction rates at 8 μg/ml (Fig. 5A). Importantly, at this dose PB more than doubled the proportion of transduced NIH-3T3 cells (Fig. 5B), suggesting that this protocol substantially increased virus infectivity on this cell type. At same conditions, protocol DT also increased transduction of all cell types (data not shown). However, protocol DT/PB was the most effective, since transduced cells exceeded 90% at day 4 (Fig 5B). Overall, the results also underlined the robustness of LAW34 under various test conditions.

### LA34 pseudotyped with RD114/TR transduces NIH-3T3 cells efficiently

**Figure 6:**
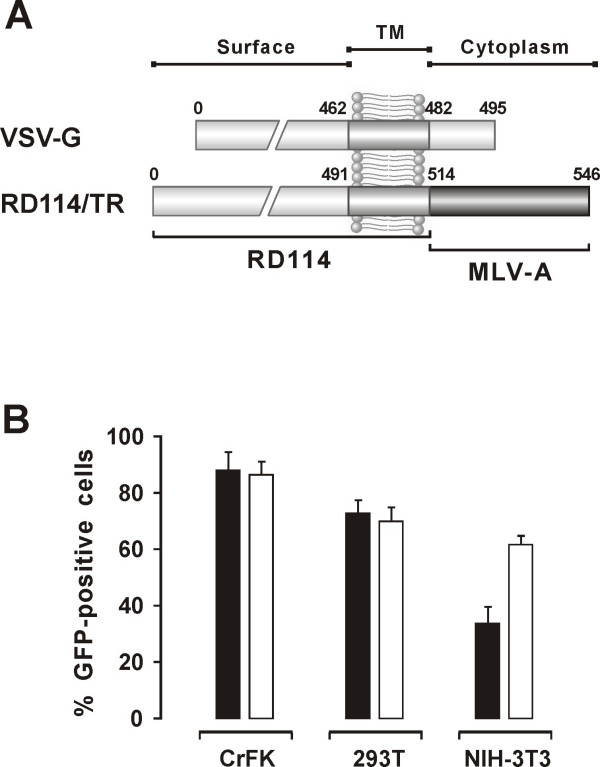
**Effect of pseudotyping LAW34 with different Env**. A) Diagrammatic representation of the VSV-G and RD114/TR glycoproteins. The latter has the extracellular and transmembrane domains of the feline endogenous retrovirus RD114 and the cytoplasmic tail of an amphotropic murine leukemia virus [36]. Numbers are aa residues. B. Transduction efficiency for the indicated cell types of LAW34 pseudotyped with VSV-G (solid columns) or RD114/TR Env (empty columns). Bars and percent GFP-positive cells evaluated as in Fig. 4.

LAW34 was pseudotyped with RD114/TR in 293T cells, and the vector thus produced (LAW34/RD114/TR; Fig. 6A) was titrated for TU in the same cell substrate using the ultracentrifugation and the PB/DT protocols. Again, in spite of a three-fold increment of the vector RNA titer after supernatant ultracentrifugation, transduction efficiency was lower compared to the PB protocol, confirming the modest performances of ultracentrifugation in our hands (Table 1). LAW34/RD114/TR and LAW34/VSV-G had essentially same number of vector RNA copies/ml, yet the former exhibited a 4 fold higher 293T transduction titer when complexed with PB, confirming the efficacy of this protocol even in the case of easy-to-transduce cells.

LAW34/RD114/TR and LAW34/VSV-G were also compared for transduction efficiency using the standard protocol, i.e. with no further manipulations or additions. Supernatants of packaging 293T cells diluted to achieve a vector RNA copy/cell ratio 1/200 of either vector performed equally in 293T and CrFK cells even after prolonged propagation (Fig. 6B and data not shown). In contrast, in NIH-3T3 cells LAW34/RD114/TR transduced with an efficiency the VSV-G counterpart had exhibited only when used with the PB protocol (Fig. 5B and 6B). Thus, LAW34/RD114/TR proved effective at transducing non feline cell lines with no need for treatments known to boost transduction efficiency.

### LAW34 transduces murine DCs and T lymphocytes efficiently

**Figure 7:**
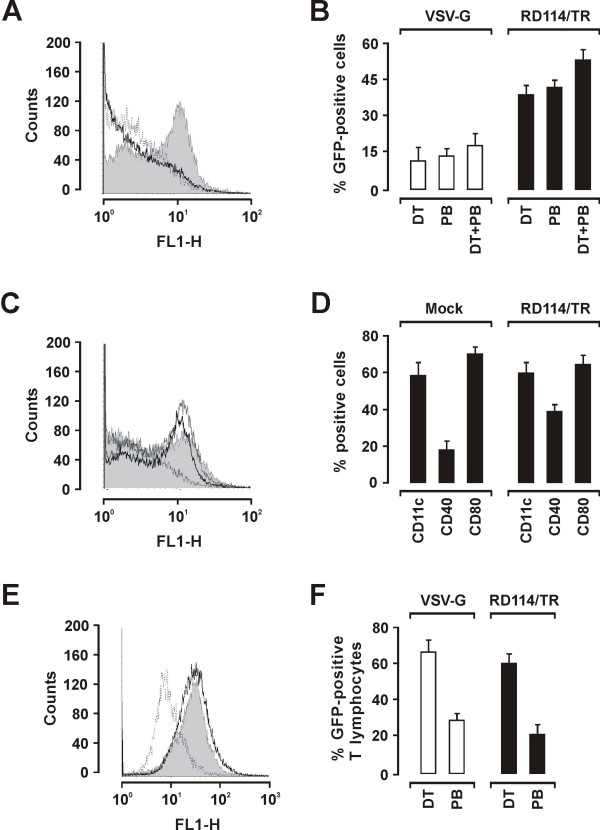
**Transduction of primary murine DCs and T lymphocytes with LAW34/VSV-G and LAW34/RD114/TR**. A) Flow cytometry analysis of DCs transduced with LAW34/VSV-G (thick line) or LAW34/RD114/TR (grey area) using PB protocol. Dotted line, untransduced, PB-treated cells. B) Efficiency of DC transduction by LAW34/VSV-G and LAW34/RD114/TR using the 3 protocols indicated. Percent GFP positive cells evaluated by flow cytometry 2 days PT. C) Intensity of GFP expression in the LAW34/RD114/TR transduced DCs in panel B, using protocols DT (thick line), PB (grey area), and DT/PB (grey line). Dotted line, untransduced DCs. D) Specific markers in DCs transduced with LAW34/RD114/TR using protocol PB or mock transduced, as examined 2 days PT. E) Flow cytometry analysis of T lymphocytes derived from murine spleen cells by ConA/IL-2 stimulation and transduced with LAW34/VSV-G (thick line) or LAW34/RD114/TR (grey area) using DT protocol. Dotted line, untransduced T lymphocytes. F) Efficiency of T lymphocyte transduction by LAW34/VSV-G and LAW34/RD114/TR using the protocols indicated.

BM-derived DCs were transduced with VSV-G or RD114/TR pseudotyped LAW34 by using 200 vector RNA copies per cell and protocols PB, DT, and DT/PB. Cells were examined 2 days later for GFP expression. LAW34-RD114/TR transduced much more efficiently than LAW34/VSV-G, regardless of protocol used. This striking difference, already clearly evident by microscopic inspection, was confirmed by flow cytometry analysis of the cells: whereas the fluorescence signal of LAW34/VSV-G transduced cells was barely distinguishable from that of mock transduced cells, LAW34/RD114/TR produced a clearly defined peak at 10^1 ^FL1-H, indicating that most DCs were transduced and actively expressing GFP (Fig. 7A). In fact, LAW34/RD114/TR performed better with all 3 protocols, transducing up to 52% DCs when the DT/PB protocol was used versus 17% with LAW/VSV-G (Fig. 7B and 7C). LAW34/RD114/TR transduction was also examined for possible effects on markers expression by DCs. DCs transduced with LAW34/RD114/TR at 200 vector RNA copies per cell using the PB protocol were compared to similarly treated cells except that the vector was replaced by the supernatant of mock-transfected cells. Relative to mock treated DCs, transduced DCs showed no changes in CD11c and CD80 expression but underwent a substantial increase of CD40-positive cells which was already evident by day 2 PT (Fig. 7D) and lasted throughout the observation period of 10 days (not shown). Of note, GFP expression by transduced cells, monitored in parallel, increased slightly over time (data not shown).

LAW34/VSV-G or LAW34/RD114/TR were also compared for ability to transduce cultured murine T lymphoblasts using the PB or the DT protocol and same vector/cell ratios. As shown by Fig. 7E and 7F, which reports the readings at day 2 PT, GFP positive cells ranged around 60% and fluorescence peaks were superimposible regardless of the Env used for pseudotyping. Also, the use of PB greatly reduced the efficiency of T lymphoblast transduction by both LAW34/VSV-G and LAW34-RD114/TR, in spite that no obvious effects on cell viability were noted. Proportions of CD4 and CD8 positive cells in the cultures remained stable for at least 10 days, following transduction (data not shown).

## Discussion

Lentivirus-derived vectors possess several advantages, including that they ensure stable and tightly controlled expression of transgenes by integrating into the cell genome, integrate preferentially into actively transcribed genes yet distantly from cellular promoters [35-37], transduce quiescent and dividing cells alike [7,30,38], and possibly have a lower insertional mutagenesis risk relative to vectors derived from other retroviruses [39]. Among such vectors, those derived from FIV have been shown to be as efficient as HIV vectors at transducing a variety of cell types and tissue compartments *in vivo *and have the added advantage of posing less safety concerns [12,28]. However, the FIV vectors described to date performed poorly when used for transducing immune cells [14-16,40], a limitation that prompted us to develop an FIV vector that might efficiently and stably deliver genes into DCs and T cells.

The vector we first constructed, LA34 is entirely derived from an FIV strain known to be much attenuated compared to field isolates [17,30] and, to further increase its safety, is self-inactivated by bearing LTRs partially deleted and totally inactive. The expression construct could be easily pseudotyped with two distinct Envs that conferred either a broad or a more restricted cell tropism. With the aim to obtain a vector that could be used in mouse models, efficiency at transducing the murine cell line NIH-3T3 was a major guiding criterion in its design as well as in optimizing transduction protocol. However, in its original format LA34 performed poorly with NIH-3T3 cells. Thus, in the attempt to overcome this drawback, the following modifications were introduced:

1) lentiviruses have evolved a PIC consisting of cellular and viral proteins which effectively delivers viral cDNA in close proximity of the cell genome. Since LA34 lacked cPPT, one of the two single-strand flaps generated during reverse transcription that are thought to optimize cDNA folding and enhance its steric fit in the nuclear pore [29], we inserted it between RRE and expression cassette. In contrast to what observed with other FIV vector formats [28,30], this modification failed to improve LA34 performances. The reason(s) was not addressed, but it is plausible that p34TF10, the parental clone from which LA34 was produced, is *per se *minimally dependent on this motif or that the p34TF10-derived cPPT we used, being slightly different in sequence from the ones previously used [30], was poorly functional.

2) the genome of lentiviruses is encapsidated through the mutual recognition of specific RNA sequences (*ψ*) and specific viral and cellular proteins [41]. In FIV, *ψ *is believed to consist of at least two discontinuous core regions, the first located upstream the major splice donor site, the second extending into *gag*. While later analyses have located the principal *ψ *domains within the first 100–120 nt [31], in early RNA protection experiments, optimal viral RNA encapsidation was obtained by extending the *gag *region to 311 nt [23]. A 310-nt sequence, that in our FIV encompasses the entire region found to be important in the above study [23], was then substituted for the 120-nt sequence present in LA34, but this brought no benefits and actually impaired nonfeline cell transduction. Again, the reason(s) for this discrepancy relative to previous studies was not investigated but it might lie in differences between the FIV clones used or in the genomic organization of the vectors developed. In any case, these results, together with the reported presence of negative regulatory sequences upstream and within *ψ *[31,42] and the consideration that fewer parental virus sequences are present in a vector the better, made us to decide in favor of the vector with the shorter *ψ*.

3) the introduction of the RNA transport element WPRE into FIV- and HIV-derived vectors has been shown to stabilize and enhance transgene expression [30,43]. When we introduced this element into LA34 we also observed a favorable impact on vector performance: the vector thus modified (LAW34) not only showed higher titers relative to LA34 but also transduced with enhanced efficiency all the cell lines. Importantly, the positive effects of WPRE were most evident in NIH-3T3 cells, suggesting that LAW34 might be particularly suitable for transducing murine cells.

We then focused on further optimizing LAW34 for usage with murine NIH-3T3 cells by modifying external coat and transduction protocol, two factors known to be as important as the vector itself for successful gene targeting and delivery. VSV-G pseudotyping is extremely convenient for vector development and *in vitro *testing since, being the cell receptor for VSV a ubiquitous phospholipid [44], it allows vector access into virtually any cell type, but this extended host range can be unpractical for vector usage *in vivo *where restricted cell targeting is often required. Moreover, VSV-G pseudotyped vectors have proved toxic for *ex vivo *cells and prone to inactivation by complement [45]. Thus, while in setting up the vector we had used VSV-G pseudotyped particles, in subsequent studies, among the many Env glycoproteins available for pseudotyping retrovirus vectors particles [46], we selected RD114/TR, derived from the feline endogenous ecotropic virus RD114, which has a sodium-dependent neutral-amino-acid transporter as cellular receptor, but having the cytoplasmatic tail of MLV-A. This chimeric Env, which in other systems has been shown to substantially increase vector titers, improve cellular localization and interaction with Gag [47], resist lysis by human complement and permit transduction of primary human lymphocytes and stem cells [24], to our knowledge had never been used with FIV vectors. The choice proved fortunate, because LAW34 pseudotyped with this Env (LAW34/RD114/TR) transduced at high efficiency all the cell lines tested and compared favorably to VSV-G pseudotyping regardless of the transducing protocol used.

We finally examined the efficiency of LAW34 at transducing *ex vivo *murine DCs and T cells using different transduction protocols. The results showed that the vector has the ability to efficiently transduce both cell types, but that the Env with which it is pseudotyped is critical for DC transduction. Indeed, while T cells were transduced to similar extents regardless of whether the vector was coated with VSV-G or RD114/TR, DCs were transduced by LAW34/RD114/TR alone, thus suggesting that previous unsatisfactorily attempts to efficiently transduce DCs with FIV vectors [14-16,40] may have been due to the use of Envs that did not permit proper entry into these cells. Of note, in agreement with findings showing that DCs transduced with different vectors develop a mature phenotype [1,6,11], murine DCs transduced with LAW34/RD114/TR exhibited at least some of the morphological and surface markers known to accompany DC maturation, thus showing that they were still functional. A further observation of interest was that LAW34 transduction of DCs and T cells occurred also in the absence of added facilitating agents, such as PB, that preferably should be avoided when vectors are used *in vivo*.

## Conclusion

The FIV-derived vector LAW34 has features compatible with a prospective usage for vaccinal purposes. These include the small size of the vector that permits to accommodate large transgenes, possibly encoding more than one protein, and the ability to deliver such transgenes into two key cell types of the immune system. *In vitro *and *in vivo *studies investigating whether transduced DCs can serve as antigen presenting cells and whether transduced T cells can be used in prime-boost vaccine experiments are currently underway.

## Competing interests

The author(s) declare that they have no competing interests.

## Authors' contributions

All Authors read and approved the final manuscript. MP is the project leader and wrote the paper, LV and AR constructed the vector and made substantial contribution to its design, FB designed and constructed the packaging vectors, FC and BDS were involved in the in vitro characterization of the vector, GF produced and analyzed the dendritic cells, MB was involved in drafting and critical revising the manuscript.
